# Glycemic control among diabetic patients in Ethiopia: A systematic review and meta-analysis

**DOI:** 10.1371/journal.pone.0221790

**Published:** 2019-08-27

**Authors:** Eyob Alemayehu Gebreyohannes, Adeladlew Kassie Netere, Sewunet Admasu Belachew

**Affiliations:** Department of Clinical Pharmacy, School of Pharmacy, University of Gondar-College of Medicine and Health Sciences, Gondar, Ethiopia; University of Oxford, UNITED KINGDOM

## Abstract

**Introduction:**

Ethiopia recorded the highest numbers of people with diabetes in Africa. It is not uncommon for diabetic patients to have poor glycemic control leading to a number of complications. The aim of this systematic review and meta-analysis is to evaluate the level of glycemic control among diabetic patients in Ethiopia by combining the studies from the existing literature.

**Materials and methods:**

The Preferred Reporting Items for Systematic Reviews and Meta-Analyses (PRISMA) guidelines was employed to plan and conduct this review. A comprehensive electronic-based literature search was conducted in the databases of MEDLINE, HINARI, GOOGLE SCHOLAR, and SCIENCEDIRECT. Open meta-analyst software was used to perform meta-analyses. Proportions of good glycemic control among diabetic patients was calculated. Odds ratio was also calculated to check the presence of statistically significant difference in glycemic control among patients with type 1 and type 2 diabetes.

**Results:**

A total of 22 studies were included in the final analysis. Meta-analysis of 16 studies showed that only one-third of patients [34.4% (95% CI: 27.9%-40.9%), p<0.001] achieving good glycemic control based on fasting plasma glucose measurements. Similar to the studies that used fasting plasma glucose, the rate of good glycemic control was found to be 33.2% [(95% CI: 21.8%-44.6%), p<0.001] based on glycosylated hemoglobin measurements. There was no statistically significant difference in the rates of glycemic control between patients with type 1 and type 2 diabetes (p = 0.167).

**Conclusion:**

High proportion of diabetic patients were unable to achieve good glycemic control. There was no difference in glycemic control among type 1 and type 2 diabetic patients.

## Introduction

Diabetes mellitus (DM) or simply diabetes is a serious, chronic disease that occurs either because of inadequate insulin production by the pancreas or inability to effectively utilize insulin by the body. It is characterized by its hallmark feature of hyperglycemia. [1, 2] It imposes an unacceptably high burden of morbidity, mortality and healthcare cost to all countries. [2] Worldwide, an estimated 422 million adults were living with diabetes in 2014. [1] An estimated 1.5 million deaths happened because of diabetes in 2012. [1] It is a disease no longer considered to affect only high-income countries. [2] The 2017 international diabetes federation estimates report a 4.4% age-adjusted relative prevalence of diabetes in Africa. Because of the large population size, Ethiopia recorded the highest numbers of people with diabetes in Africa with an estimated 2.6 million diabetic patients. [2]

There are different types of diabetes. The widely accepted classification of diabetes includes type 1 diabetes, type 2 diabetes, and gestational diabetes. There are also other specific causes of diabetes. [2] Uncontrolled long-term hyperglycemia can result in damage to various parts of the body leading to several macrovascular and microvascular complications such as neuropathy, nephropathy, retinopathy, cardiovascular diseases, amputations, and even premature death. [1, 2] More than two-fifth of all hyperglycemia-associated deaths occur among patients younger than 70 years. [1] Cardiovascular disease (CVD) is a more common cause of death in diabetic patients. [3]

Several studies reported that more than half of diabetic patients have poor glycemic control [4, 5, 6]. As there are a number of complications that may result from poor glycemic control, patients should strive to achieve predefined glycemic goals. [2, 3] A reasonable A1C goal for many adults is 7%. However, less stringent (A1C of 8%) or more stringent (eg A1C of 6.5%) goals might be beneficial for some patients. Other glycemic goals include preprandial capillary plasma glucose 80–130 mg/dL and peak postprandial capillary plasma glucose <180 mg/dL. For children and adolescents, the A1C goal is 7.5% but can be reduced if it can be achieved without a significant burden. The preprandial and bedtime goals include 90–130 mg/dL and 90–150 mg/dL, respectively. [3] A number of studies that assessed glycemic control among Ethiopian patients have been published. However, most of these studies are single-centered and with relatively small sample sizes. This makes it difficult for policymakers to make decisions based on such studies. Therefore, the aim of this systematic review and meta-analysis is to evaluate the level of glycemic control among diabetic patients in Ethiopia by combining the studies from the existing literature.

## Materials and methods

The Preferred Reporting Items for Systematic Reviews and Meta-Analyses (PRISMA) guidelines [7] was employed to plan and conduct this review (S1 Table).

### Search strategy and review process

**Fig 1:**
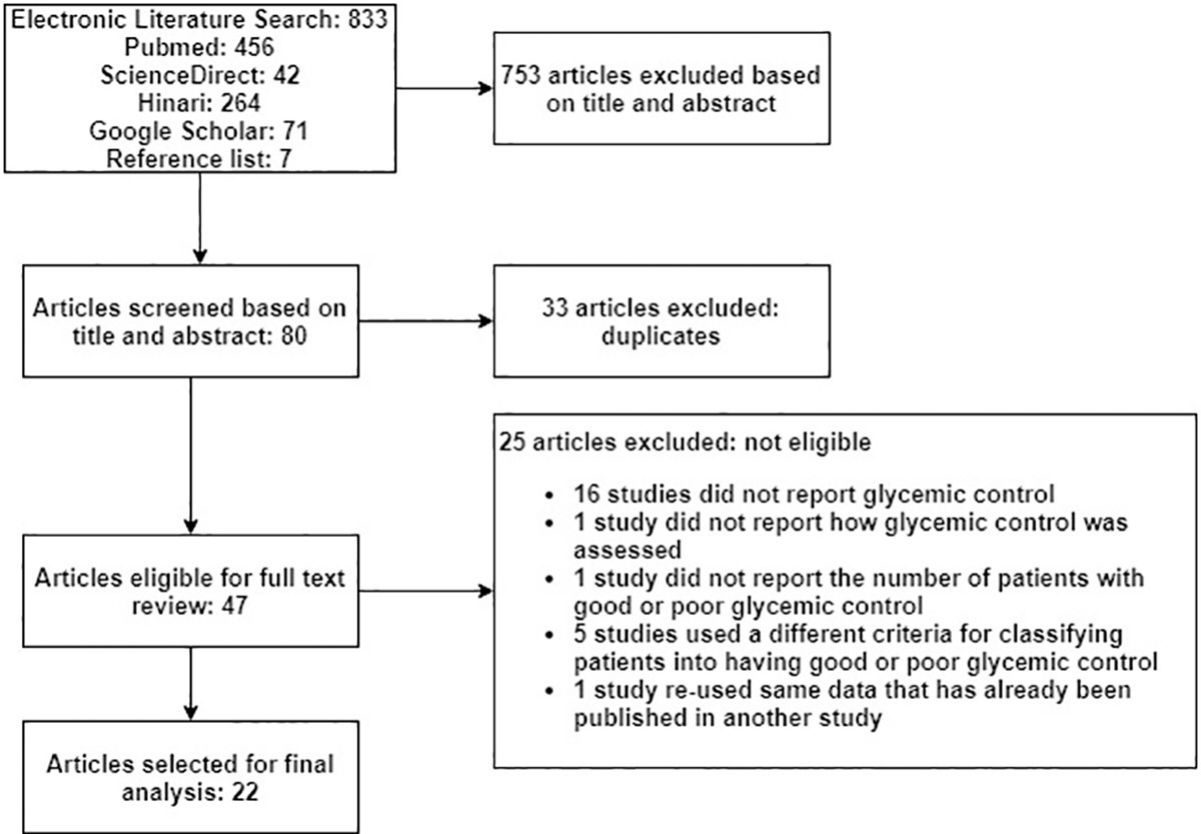
Flow diagram showing systematic literature search.

A comprehensive electronic-based literature search was conducted in the databases of MEDLINE, HINARI, GOOGLE SCHOLAR, and SCIENCEDIRECT by one of the authors (EAG). The literature search was further strengthened by searching relevant articles from the reference list of retrieved articles (AKN). During searching the following search terms were alternatively combined using the Boolean logic (AND/OR): diabetes mellitus, diabetes, diabetic, glycemic control, glucose, glycemia, blood sugar, A1C, and Ethiopia: ((diabetes mellitus OR diabetes OR diabetic OR glycemic control OR glucose OR glycemia OR blood sugar OR a1c)) AND Ethiopia. All searches were conducted in July 2018. Two of the reviewers (EAG and AKN) screened the titles and abstracts of each article to identify potentially eligible studies. After removing duplicates, EndNote X5 (Thomson Reuters, USA) was used to create a bibliographical database of the retrieved references. Then, EAG and SAB independently extracted relevant data from full-length articles that fulfilled the inclusion criteria (Fig 1). Discrepancies were resolved by mutual consent after discussion and independent review from the third researcher (AKN).

### Inclusion and exclusion criteria

Papers fulfilling the following criteria were included in the study: studies presented as original articles; studies that assessed glycemic control among diabetic patients using one or more of the following: an FPG level (of <126 mg/dl, <130 mg/dl, or ≤130 mg/dl) and/or an A1C level (of <7% or ≤7%); studies conducted in Ethiopia; studies conducted in the past 15 years; and studies written in English. The following papers were excluded from the study: studies that did not explain the criteria for good or poor glycemic control; studies that didn’t state the number of patients with poor or good glycemic control; studies that used an A1C cut-off point other than 7% for adults and an FPG cut-off point other than 126 or 130 mg/dl (S2 Table). Studies published between 7 August 2008 and 25 July 2018 were included in the present study.

### Data extraction

Data on the year of publication, study design, length of study, and geographic location of the study area, participants’ mean age and ranges, and sex of study participants were extracted. Data regarding clinical and laboratory characteristics (the type of diabetes, mean duration since diagnosis of diabetes, mean FPG, and mean A1C), treatment options, medication adherence, glycemic control, and complications were also extracted.

### Quality assessment and sensitivity analysis

Quality assessment was performed using the 22-items “STrengthening the Reporting of OBservational studies in Epidemiology” (STROBE) checklist [8]. Studies that scored more than 75% (17 out of the 22 items) were considered to have good quality (S3 Table). Studies that used FPG to measure glycemic control had 2 cut-off points, 126 mg/dl and 130 mg/dl. To address this issue, a sensitivity analysis was performed between these two groups of patients. In addition, sensitivity analysis was also done based on study quality.

### Operational definitions

Patients with an FPG level of <126 mg/dl, <130 mg/dl, or ≤130 mg/dl or patients with an A1C level of <7% or ≤7% were considered to have good glycemic control. On the other hand, patients with an FPG level of ≥126 mg/dl, >130 mg/dl, or ≥130 mg/dl or patients with an A1C level of >7% or ≥7% were considered to have poor glycemic control.

### Statistical analysis

Open meta-analyst software (www.cebm.brown.edu/openmeta) was used to perform meta-analyses of proportions of good glycemic control among diabetic patients based on the above thresholds. Proportions were used to determine the level of glycemic control. The odds ratio was calculated to check the presence of a statistically significant difference in glycemic control among patients with type 1 and type 2 diabetes. Because of high levels of heterogeneity in terms of population and treatment, a random effect model was used to perform all meta-analyses. To assess publication bias Comprehensive Meta-Analysis software version 3 was used. Funnel plots, Egger’s test, and Begg-Mazumdar tests were used to check for publication bias.

## Results

### Patient and study characteristics

A search of the four databases and reference list of included studies yielded 840 titles, of which 22 studies [9–30] fulfilled the inclusion criteria (Fig 1). All of the studies were cross-sectional, were conducted in one or more health institutions, and had sample sizes ranging from 86 [23] to 544 patients [19]. Except for one multicenter study [28], all of the studies included in the final analyses were from single institutions [9–27, 29, 30]. The studies were conducted in 4 regions including Oromia (9 studies) [9–15, 29, 30], Addis Ababa (5 studies) [16–20], Amhara (4 studies) [21–24], and Tigray (4 studies) [25–28] (Table 1). Though 3285 male and 2460 female patients were included in the studies, data on glycemic control was available for a total of 5719 patients. This is because FPG was not done for 9 and 17 patients in the Hailu et al [13] and Gudina et al [14] studies, respectively. These 5719 patients were included in the final analyses of which 20 studies [9, 10, 12–25, 27–30] reported the mean age of the participants. Accordingly, the mean age of the participants was 45.19 years. Most of the studies included adult patients (15 years or older) [9–17, 20–30] while only 2 studies [18, 19] included children younger than 15 years. Because Abebe et al 2014 [24] and Abebe et al 2015 [31] studies are identical with the same patient populations and the same outcomes, we opt to use only one of these two studies (Abebe et al 2014) [24].

**Table 1:** Overview of studies conducted in Ethiopia from (N = 5719).

Author and year of publication	Journal	Location	Study duration (Months)	DM type	Sample size	Mean age	Treatment options	Criteria for good glycemic control
Fiseha et al 2018	BMC Res Notes	Dessie	4	Type 1 (N = 126) and 2 (N = 258)	384	45	Insulin; oral agents	FPG≤130
Mideksa et al 2018	Adipocyte	Mekelle	2	Type 1 (N = 72) and 2 (N = 264)	336	49.01	Insulin; oral agents	A1C<7; FPG <130
Shimels et al 2018	Ethiop J Health Sci	Addis Ababa	3	Type 2	361	54.8	Insulin; oral agents	FPG<130
Tekalegn et al 2018	PLoS One	Addis Ababa	2	Type 2	412	52	Insulin; oral agents	FPG≤130
Tsadik et al 2018	J Diabetes Res	Addis Ababa	4	Type 1	176	11.36	Insulin	A1C<7.5
Belay et al 2017	Int J Chronic Dis	Mekelle	5	Type 2	188	Not stated	Insulin; oral agents	FPG<130; A1C<7
Mariam et al 2017	J Diabetes Res	Gondar	2	Type 1 (N = 110) and 2 (N = 169)	279	49.8	Insulin; oral agents	FPG<126
Muleta et al 2017	Clin Hypertens	Jimma	1	Type 2	131	50.69	Insulin; oral agents	FPG<130
Seid et al 2017	BMC Endocr Disord	Multi-center	2	Type 1 (N = 128) and 2 (N = 84)	212	43.39	Not stated	FPG<126
Cheneke et al 2016	BMC Res Notes	Jimma	3	*Type 1 and 2	148	48.5	Insulin; oral agents	A1C<7
Kassahun et al 2016	BMC Res Notes	Jimma	3	Type 2	309	Not stated	Insulin; oral agents	FPG≤130
Shibeshi et al 2016	BMC Res Notes	Addis Ababa	Not stated	Type 1	86	13.7	Insulin	A1C<10; FPG<150 (age ≤11), FPG<130 (age 12–15), FPG<120 (age≥16)
Alemu et al 2015	Diabetes Res Clin Pract	Gondar and surrounding	Not stated	Type 1	544	34.47	Insulin	A1C≤7
Abebe et al 2014	Springerplus	Gondar	2	Type 1 (N = 111) and 2 (N = 280)	391	50.4	Insulin; oral agents	A1C<7
Asfaw et al 2014	Arch Pharm Pract	Addis Ababa	1	Type 2	103	52.2	Insulin; oral agents	FPG<126
Woldu et al 2014	Endocrino Metab Synd	Ambo	6	Type 2	102	51.75	Insulin; oral agents	FPG<126
Angamo et al 2013	PLoS One	Jimma	3	Type 1 (N = 163) and 2 (N = 121)	284	41.37	Insulin	FPG<126
Hailu et al 2012	Afr J Prim Health Care Fam Med	Jimma	1	*Type 1 and 2	333	45.2	Insulin; oral agents	FPG<126
Teklay et al 2013	J Med Sci	Jimma	3	Type 2	267	52.4	Insulin; oral agents	FPG≤130
Gudina et al 2011	BMC Endocr Disord	Jimma	2	*Type 1 and 2	312	48.4	Insulin; oral agents	FPG≤130
Tamiru et al 2010	Ethiop J Health Sci.	Jimma	3	Type 1 (N = 85) and 2 (N = 171)	256	45.3	Insulin; oral agents	FPG<126
Gill et al 2008	QJM	Mekelle	1.5	Type 1 (N = 42) and 2 (N = 63)	105	41	Insulin; oral agents	A1C≤7

*Unable to determine the number of patients with type 1 and type 2 DM.

Majority of the studies included patients with type 2 diabetes (N = 3706 vs N = 1891, unknown for the remaining 148 patients). Twelve studies [12–16, 21, 23–28] reported a mean duration of diabetes since the initial diagnosis which ranged from 5 years [12] to 7.83 years [25]. Eight studies [12, 14, 16, 18–20, 23, 27] reported the specific antidiabetic medications used while another 13 studies [9–11, 13, 15, 17, 21, 22, 24–26, 29, 30] reported only the number of patients on insulin treatment without mentioning the specific oral antidiabetic medication used. According to the first eight studies, from a total of 2480 patients, 1530, 509 and 441 patients used insulin, metformin, and glibenclamide alone or in combination with other antidiabetic medications, respectively. On the other hand, of the 11 studies who mentioned only insulin use, 1585 of 3545 patients used insulin alone or in combination with other antidiabetic medications. Three studies [9, 11, 23] reported adherence to antidiabetic medications using an 8-items Morisky’s medication adherence scale (N = 830) [9, 11, 24]. A meta-analysis of these three studies indicated that medication adherence was good in more than half of patients [52.2% (33.4%-71.0%), p<0.001].

### Glycemic control

**Fig 2:**
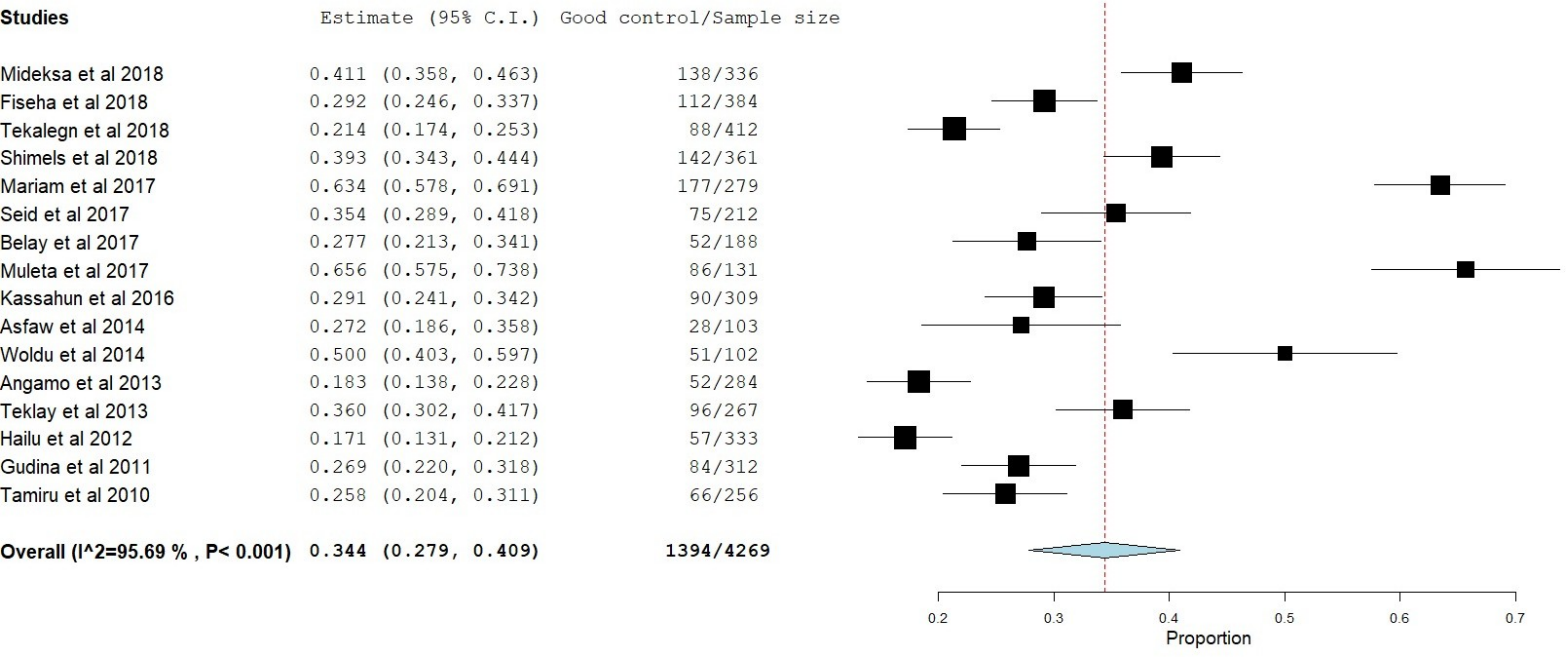
Proportion of patients with good glycemic control based on FPG measurements.

**Fig 3:**
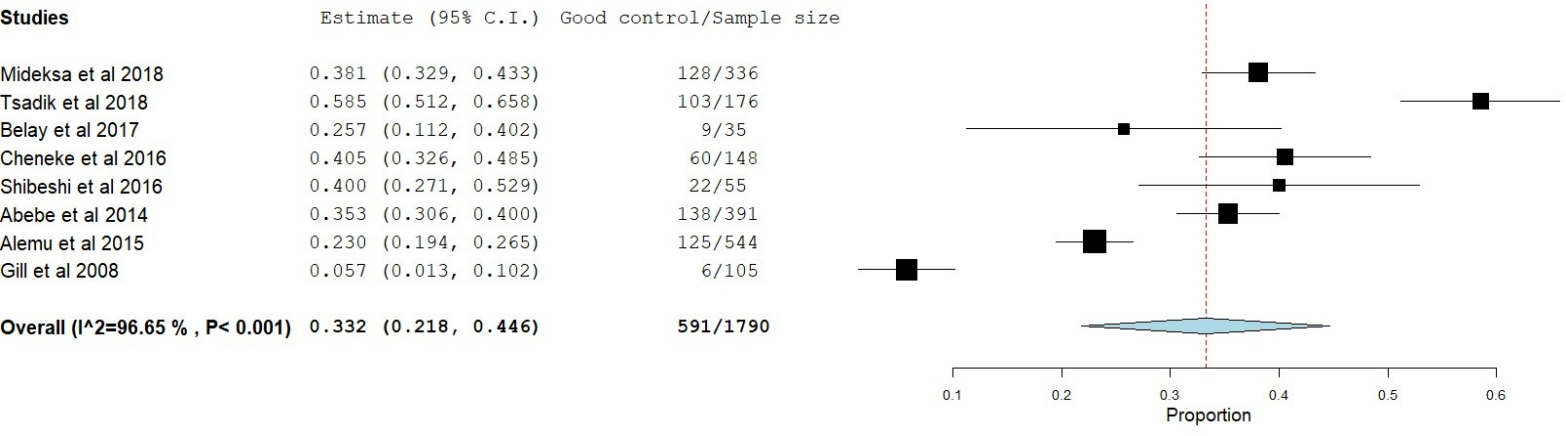
Proportion of patients with good glycemic control based on A1C measurements.

Glycemic control was assessed in two different ways, FPG and A1C. Sixteen studies [9, 11–17, 20–22, 25, 26, 28–30] reported glycemic control using FPG. Twelve studies [12–14, 16, 17, 20, 21, 25, 27, 28–30] reported mean FPG levels ranging from 154 mg/dl [16] to 216 mg/dl [27]. Based on these 12 studies, the mean FPG level was 170.57 mg/dl. Seven studies [12, 13, 15, 20, 22, 28, 30] used an FPG level of 126 mg/dl as a cut-off point for good/poor glycemic control while the remaining 9 studies [9, 11, 14, 16, 17, 21, 25, 26, 29] used 130 mg/dl. According to these 16 studies, only one-third of the patients [34.4% (95% CI: 27.9%-40.9%), p<0.001] were able to achieve good glycemic control (Fig 2). On the other hand, eight studies [10, 18, 19, 23–27] reported the rates of good glycemic control using A1C. Most of these studies [10, 23–27] used an A1C cut-off point of 7% while two pediatric studies [18, 19] used different cut-off points based on the patients’ age. Similar to the studies that used FPG, the rate of good glycemic control was found to be 33.2% [(95% CI: 21.8%-44.6%), p<0.001] (Fig 3).

The current study tried to compare glycemic control among patients with type 1 and type 2 diabetes. The proportion of good glycemic control was found to be comparable between these two groups of patients (Table 2).

**Table 2:** Glycemic control among patients with type 1and type 2 diabetes.

	*Type 1 diabetes*				*Type 2 diabetes*			
	*Studies*	*Proportion (95% CI)*	*p-value*	*I*^*2*^ *(%)*	*Studies*	*Proportion (95% CI)*	*p-value*	*I*^*2*^ *(%)*
A1C	4 studies (19, 20, 24, 25)	31.0% (14.2–47.8%)	<0.001	96.46	2 studies (25, 27)	35.5% (19.2–51.7%)	0.035	77.53
FPG	2 studies (13, 15)	20.7% (15.8–25.5%)	0.583	0	10 studies (10, 12, 13, 15, 17, 18, 21, 27, 30, 31)	33.9% (26.3–41.5%)	<0.001	93.64

Two studies [13, 15] compared treatment outcomes among patients with type 1 and type 2 diabetes using FPG. A meta-analysis of these two studies showed that there is no difference in good glycemic control between these two groups of patients [OR (95% CI) = 1.098 (0.630–1.913), *p* = 0.167].

### Predictors of glycemic control

Several predictors of glycemic control were reported by different studies. Younger age [10, 12], male sex [10], being married (vs single) [10], and living in rural areas [21] were all associated with poor glycemic control. Longer duration of diabetes [10, 17, 21, 26] and insulin-induced lipohyperthrophy [18] were also associated with poor glycemic control. The presence of complications indicated poor glycemic control [12]. Conflicting results were reported on the effect of insulin monotherapy on glycemic control when compared to the combination of insulin and oral antidiabetic medications. While Chenek et al [10] reported better glycemic control of insulin over the combination treatment, Fiseha et al [21] reported to the contrary. Patients who were on monotherapy with oral antidiabetic medications had better glycemic control than insulin monotherapy [14, 17] and the combination of two oral antidiabetic medications [13, 14, 25]. Three studies [11, 14, 25] reported that patients with oral antidiabetic medication monotherapy had better glycemic control when compared to combination of insulin with oral antidiabetic medications. On the other hand, Fiseha et al [21] reported that patients who were on combination oral antidiabetic agents had better glycemic control than those on monotherapy. Other predictors of poor glycemic control include non-adherence to dietary plan [16] and medications [11, 24], poor knowledge of patients about the disease and its management [12, 13], and lower level of education [10, 11, 13, 21].

### Microvascular complications

Eight [10, 11, 14, 16, 19, 22, 27, 30], 4 [10, 11, 19, 27] and 7 [9, 11, 14, 19, 23, 26, 27] studies reported the proportion of patients who experienced diabetic neuropathy (272/1702), nephropathy (82/648) and retinopathy (225/1675), respectively. Accordingly, the most frequent complication of the three was diabetic neuropathy [15.0% (7.5%-22.4%), p<0.001] followed by retinopathy [12.2% (6.2%-18.3%), p<0.001] and nephropathy [8.6% (1.8%-15.5%), p<0.001].

### Sensitivity analysis and quality assessment

Because the studies that used FPG to measure glycemic control used two different cut-off points, sensitivity analysis was conducted to check if there is any difference between studies that used an FPG of 126 mg/dl [12, 13, 15, 20, 22, 28, 30] and 130 mg/dl [9, 11, 14, 16, 17, 21, 25, 26, 29]. Accordingly, good glycemic control in studies that used an FPG cut-off point of 130 mg/dl and 126 mg/dl was found to be 34.8% [(95% CI: 28.0%-41.7%), p<0.001, I^2^ = 93.52)] and 33.7% [(95% CI: 20.6%-46.9%), p<0.001, I^2^ = 97.28], respectively.

A sensitivity analysis was also performed by excluding studies deemed to have poor quality. Quality assessment was performed using the STROBE statement [8]. Of the 22 studies included in the analysis, 20 [9–19, 21–26, 28–30] and 2 [20, 27] studies were found to have high and low quality, respectively. Since, the Gill et al [27] and Asfaw et al [20] studies were deemed to have poor quality, they were excluded in performing meta-analyses using A1C and FPG measurements, respectively. Accordingly, 37.4% [(95% CI: 28.2%-46.6%), p<0.001, I^2^ = 93.19%] and 34.8% [(95% CI: 28.0%-41.6%), p<0.001, I^2^ = 95.97%] of patients had good glycemic control based on A1C (S1 Fig) and FPG (S2 Fig) measurements, respectively.

**Fig 4:**
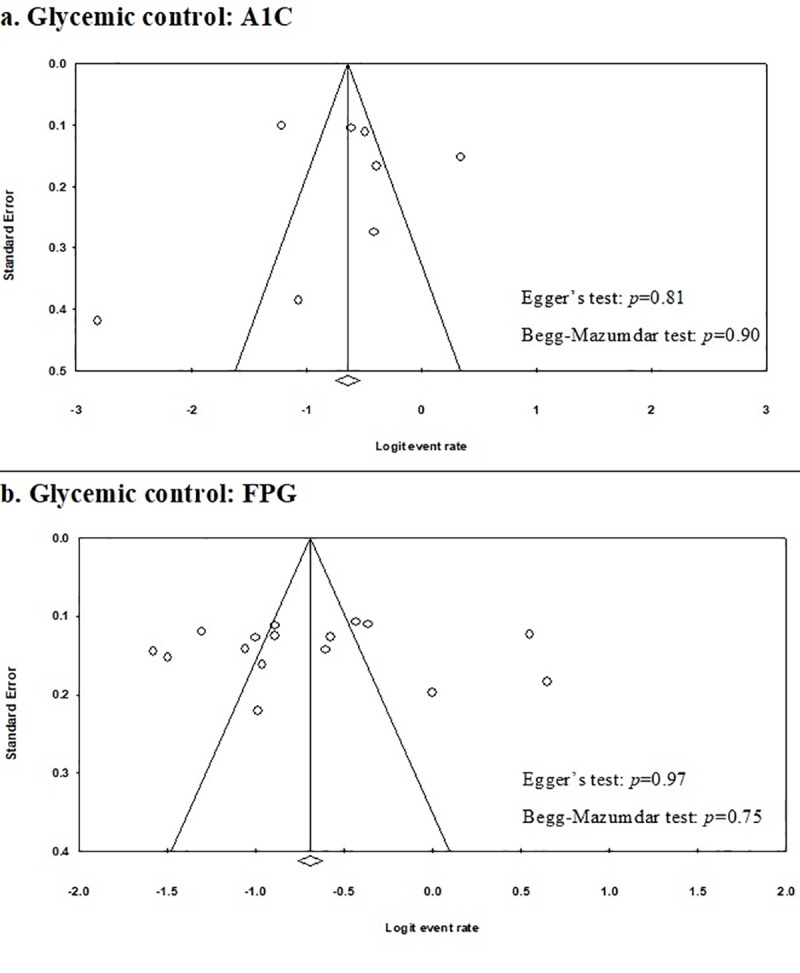
Funnel plots showing publication bias: a. studies that reported glycemic control using A1C; b. studies that reported glycemic control using FPG.

A funnel plot was used to assess publication bias (Fig 4). Statistical tests failed to show evidence of publication bias for studies that used FPG (Egger’s test, P = 0.97; Begg-Mazumdar test, P = 0.75), as well for A1C (Egger’s test, P = 0.81; Begg-Mazumdar, P = 0.90) as a measure of glycemic control.

## Discussion

This systematic review and meta-analysis was conducted primarily to determine the overall estimates of poor and good glycemic control in Ethiopia based on 22 eligible studies [9–30]. Thus, glycemic control was assessed using two parameters, FPG and A1C. Based on A1C measurements, only one-third (33.2%) of the diabetic patient was found to have good glycemic control. A similar proportion of patients (34.4%) also were able to achieve their FPG targets. This indicates that a large proportion of diabetic patients (65.6–66.8%) were not able to achieve good glycemic control which is slightly higher than a study conducted in Riyadh, Saudi Arabia [4]. The proportion of diabetic patients with poor glycemic control in Riyadh (A1C≥7%) was 60.3% [4]. Likewise, glycemic control in Ethiopia was poorer than a study done in Lusaka, Zambia [5]. In this study, 61.3% of diabetic patients failed to achieve good glycemic control which was lower than the present study by 5.5%. In addition, the Lusaka study used A1C≥6.6% as a cut-off point for poor glycemic control which may have overestimated the proportion of poor glycemic control. However, the results of the present study showed better glycemic control than another African study done in three urban clinics of Kampala, Uganda [6]. The proportion of patients with poor glycemic control (A1C≥7%) in the Kampala study was reported to be 73.5% which was higher than the present study by 6.7%.

The present study also tried to conduct a sub-group analysis based on the type of diabetes. Only a couple of studies [12, 14] compared glycemic control among patients with type 1 and type 2 diabetes and failed to show any difference (*p* = 0.167). On the other hand, in the present study, a meta-analysis of studies that independently assessed glycemic control only in either type of diabetes showed that glycemic control was better among type 2 diabetic patients than those of type 1 diabetic patients. The proportion of type 2 diabetic patients with good glycemic control based on A1C and FPG measurements were 35.5% (vs 31.0%) and 33.9% (vs 20.7%), respectively.

Type 2 diabetic patients accounted for two-third (66.21%) of all diabetic patients in the present study. Most other studies also focused on glycemic control in type 2 diabetic patients. Accordingly, based on A1C measurements, glycemic control among type 2 diabetic patients in the present study was better (33.4%) than studies conducted in Eastern Sudan (28.1%) [32], Saudi Arabia (25.1%) [33] and Palestine (19.5%) [34]. However, glycemic control in the present study was lower than one European study where the proportion of type 2 diabetic patients with ≥7% was 36% [35]. It was even far lower than a Chinese study by Li et al [36] where 49.7% and 42.7% of type 2 diabetic patients achieved good glycemic control based on A1C and FPG measurements, respectively.

Predictors of glycemic control were also assessed in the present study. Younger age was identified as a predictor of poor glycemic control in Ethiopia. On the other hand, a Palestinian study reported older age as a predictor of good glycemic control [34]. Contrary to the findings of the present study, previous studies reported that male sex was associated with either better glycemic control [34] or no association at all [35]. Living in rural areas was found to be associated with poor glycemic control in the present study. This is most likely because of lower health literacy in patients from rural areas as good health literacy is associated with better glycemic control in the present study as well as in previous studies [34, 37].

Insulin-induced lipohyperthrophy was associated with poor glycemic control. In addition, patients with longer duration of diabetes had poor glycemic control in the present study. Similarly, several studies support this claim [33–36]. As diabetes is a progressive disease, the response to intensive glucose control declines through time making it difficult to achieve good glycemic control [38]. Similar to the present study, level of adherence were also mentioned as predictors of glycemic control where people with low-level adherence tend to have poor glycemic control and those with a higher level of adherence have good glycemic control [5, 34]. Previous studies reported that insulin therapy [6, 35] and metformin monotherapy [6] were associated with poor glycemic control. However, studies included in the present review reported conflicting results. This might be because of the fact that those with poorer glycemic control may require combination treatment and insulin therapy.

As the proportion of diabetic patients with poor glycemic control is high, different efforts should be implemented in the country so as to improve glycemic control. Poor medication adherence was identified as a predictor of poor glycemic control and interventions that improve medication adherence can have a positive impact on glycemic control. One study conducted in Ethiopia pointed out that the involvement of pharmacists in the management of diabetes improves adherence to antidiabetic medications [39]. Other effective interventions that have proven to be effective in improving glycemic control elsewhere include self-monitoring blood glucose [40, 41], encouraging patients on spending less time sitting and exercising more [42, 43], peer support [44], and psychological interventions [45, 46].

Even though the quality of a couple of the studies [20, 27] was of “poor” quality based on the quality assessment tool used [8], this did not significantly affect the quality of the meta-analysis. This is because we primarily needed the proportion of patients with good or poor glycemic control and this has been consistently reported throughout the 22 studies.

### Strengths and limitations

This systematic review and meta-analysis has several strengths. It is the first study that combined results of several studies in the country giving stronger evidence on the status of glycemic control. It was able to include glycemic control of a relatively large number of patients (N = 5719) which is much more than sample sizes of individual studies. It also tried to compare glycemic control among type 1 and type 2 diabetic patients. Most importantly, the study analyzed results based on A1C and FPG measurements which makes the results more valid. Despite its strengths, the study also has few limitations. Though most of the studies are of good quality, all of the studies included in the analysis were cross-sectional. In addition, the study was not able to conduct sub-group analysis based on the different treatment options which would have given important information to identify a potential target in order to improve glycemic control.

## Conclusions

In conclusion, this systematic review and meta-analysis revealed that a high proportion of diabetic patients were unable to achieve good glycemic control. This has contributed to the frequent occurrence of diabetic complications such as neuropathy, retinopathy, and nephropathy. There was no difference in glycemic control among type 1 and type 2 diabetic patients. A number of factors contributed to the high proportion of poor glycemic control. These include poor medication adherence, low level of education, low level of health literacy, and lipoatrophy at insulin injection sites.

## Supporting information

S1 Table PRISMA checklist. (DOCX)

S2 Table Excluded studies after review of full-text articles. (DOCX)

S3 Table Quality assessment of included studies using the STROBE checklist. (DOCX)

S1 Fig Sensitivity analysis of glycemic control based on the quality of studies (using A1C measurements). (TIF)

S2 Fig Sensitivity analysis of glycemic control based on the quality of studies (using FPG measurements). (TIF)
