# Supplementary material for: Glycemic control among diabetic patients in Ethiopia: A systematic review and meta-analysis
Source: PLoS One. 2019 Aug 27;14(8):e0221790. doi: 10.1371/journal.pone.0221790 (PMC6711596; doi:10.1371/journal.pone.0221790)
Supplement: S2 Table — (DOCX) [file pone.0221790.s002.docx]

**S2_Table. Excluded studies after review of full text articles**

|  | **Author and year of publication** | **Title** | **Journal** | **Reason** |
| --- | --- | --- | --- | --- |
| 1 | Birara et al 2018 | Metabolic syndrome among type 2 diabetic patients in Ethiopia: a cross-sectional study | BMC Cardiovasc Disord | Did not report glycemic control |
| 2 | Chisha et al 2018 | Incidence and factors associated with diabetic retinopathy  among diabetic patients at Arbaminch General Hospital, Gamo Gofa Zone (Longitudinal Follow Up Data Analysis) | J diabetolo | Did not report glycemic control |
| 3 | Gebreyes et al 2018 | Prevalence of high blood pressure, hyperglycemia, dyslipidemia, metabolic syndrome and their determinants in Ethiopia: evidences from the national NCDs STEPS  survey, 2015 | PLoS One | Did not report glycemic control |
| 4 | Alemayehu et al 2017 | Prevalence of diabetes mellitus in 7– 12 years old school children presenting to department of pediatrics and child health, Tikur Anbessa specialized teaching hospital, Addis Ababa, Ethiopia | Ethiop Med J | Did not report glycemic control |
| 5 | Atikilt et al 2017 | Clinical characteristics of diabetic ketoacidosis in children with newly diagnosed type 1 diabetes in Addis Ababa, Ethiopia: a cross-sectional study | PLoS One | Did not report glycemic control |
| 6 | Berihun et al 2017 | Correlates of time to microvascular complications among diabetes mellitus patients using parametric and non-parametric approaches: a case study of Ayder referral hospital, Ethiopia. | Ethiop. J. Sci. & Technol | Did not report glycemic control |
| 7 | Chisha et al 2017 | Prevalence and factors associated with diabetic retinopathy among diabetic patients at Arbaminch General Hospital, Ethiopia:Cross sectional study | PLoS One | Did not report glycemic control |
| 8 | Erku et al 2017 | The impact of pharmacist-led medication therapy management on medication adherence in patients with type 2 diabetes mellitus: a randomized controlled study | Pharm Pract | Did not report glycemic control |
| 9 | Hintsa et al 2017 | Determinants of diabetic nephropathy in Ayder Referral Hospital, Northern Ethiopia: A case-control study | PLoS One | Did not explain how glycemic control was assessed |
| 10 | Mulatu et al 2017 | Pattern of antihypertensive treatment and blood pressure control among diabetic outpatients in Addis Ababa, Ethiopia | Journal of Diabetes and Metabolism | Did not report how many patients had good or poor glycemic control |
| 11 | Woyesa et al 2017 | Hyperuricemia and metabolic syndrome  in type 2 diabetes mellitus patients at  Hawassa university comprehensive  specialized hospital, South West Ethiopia | BMC Endocr Disord | Used a different FPG criteria (110 mg/dl) |
| 12 | Yigazu et al 2017 | Glycemic control and associated factors among type 2 diabetic patients at Shanan Gibe Hospital, Southwest Ethiopia | BMC Res Notes | Used a different FPG criteria (70-130 mg/dl) |
| 13 | Biadgo et al 2016 | Hematological indices and their correlation with fasting blood glucose level and anthropometric measurements in type 2 diabetes mellitus patients in Gondar, Northwest Ethiopia | Diabetes Metab Syndr Obes | Did not report glycemic control |
| 14 | Abebe et al 2015 | Level of sustained glycemic control and associated factors among patients with diabetes mellitus in Ethiopia: a hospital-based cross-sectional study | Diabetes Metab Syndr Obes | Data split from another study (Abebe et al 2015) |
| 15 | Abejew et al 2015 | Diabetic complications among adult diabetic patients of a tertiary hospital in Northeast Ethiopia | Adv Public Health | Did not report glycemic control |
| 16 | Ambachew et al 2015 | Dyslipidemia among diabetic patients in Southern Ethiopia: Cross-sectional study | J Diabetes Endocrinol | Used a different FPG criteria (120 mg/dl) |
| 17 | Bekele et al 2015 | Assessment of liver function tests and associated risk factors among diabetic patients attending diabetes clinic of Jimma University Specialized Hospital, Jimma, Ethiopia | J Pharm Biomed Sci | Did not report glycemic control |
| 18 | Desse et al 2015 | Predictors and treatment outcome  of hyperglycemic emergencies at Jimma University Specialized Hospital, southwest Ethiopia | BMC Res Notes | Did not report glycemic control |
| 19 | Gebrekirstos et al 2013 | Prevalence and factors associated with diabetic foot ulcer among  adult patients in Ayder referral hospital diabetic clinic Mekelle, North Ethiopia, 2013 | Journal of Diabetes and Metabolism | Did not report glycemic control |
| 20 | Mullugeta et al 2012 | Dyslipidemia associated with poor glycemic control in type 2  diabetes mellitus and the protective effect of metformin  supplementation | Ind J Clin Biochem | Used a different A1C criteria (8%) |
| 21 | Nebeck et al 2012 | Hematological parameters and metabolic syndrome: Findings from an occupational cohort in Ethiopia | Diabetes Metab Syndr | Did not report glycemic control |
| 22 | Wabe et al 2011 | Medication adherence in diabetes mellitus and self-management practices among type-2 diabetics in Ethiopia | N Am J Med Sci | Used a different FPG criteria (90-100 mg/dl) |
| 23 | Worku et al 2010 | Patterns of diabetic complications at Jimma University Specialized hospital, Southwest Ethiopia | Ethiop J Health Sci | Did not report glycemic control |
| 24 | Siraj et al 2006 | Lipid and lipoprotein profiles in Ethiopian patients with diabetes mellitus | Metabolism | Did not report glycemic control |
| 25 | Gebre-yohannes et al 1997 | Glycaemic control and its determinants in diabetic patients in Ethiopia | Diabetes Res Clin Pract | A very old study |
