# Supplementary material for: Glycemic control among diabetic patients in Ethiopia: A systematic review and meta-analysis
Source: PLoS One. 2019 Aug 27;14(8):e0221790. doi: 10.1371/journal.pone.0221790 (PMC6711596; doi:10.1371/journal.pone.0221790)
Supplement: S3 Table — (DOCX) [file pone.0221790.s003.docx]

**S3_Table. Quality assessment of included studies using the STROBE checklist**

|  | **Study** | **Score (out of 22)** | **Score (percentage)** | **Quality** |
| --- | --- | --- | --- | --- |
| 1 | Fiseha et al 2018 | 21 | 95.5% | High |
| 2 | Mideksa et al 2018 | 19 | 86.4% | High |
| 3 | Shimels et al 2018 | 17 | 77.3% | High |
| 4 | Tekalegn et al 2018 | 20 | 90.9% | High |
| 5 | Tsadik et al 2018 | 18 | 81.8% | High |
| 6 | Belay et al 2017 | 18 | 81.8% | High |
| 7 | Mariam et al 2017 | 21 | 95.5% | High |
| 8 | Muleta et al 2017 | 21 | 95.5% | High |
| 9 | Seid et al 2017 | 21 | 95.5% | High |
| 10 | Cheneke et al 2016 | 20 | 90.9% | High |
| 11 | Kassahun et al 2016 | 22 | 100% | High |
| 12 | Shibeshi et al 2016 | 19 | 86.4% | High |
| 13 | Alemu et al 2015 | 18 | 81.8% | High |
| 14 | Abebe et al 2014 | 17 | 77.3% | High |
| 15 | Asfaw et al 2014 | 15 | 68.2% | Low |
| 16 | Woldu et al 2014 | 20 | 90.9% | High |
| 17 | Angamo et al 2013 | 20 | 90.9% | High |
| 18 | Teklay et al 2013 | 21 | 95.5% | High |
| 19 | Hailu et al 2012 | 22 | 100% | High |
| 20 | Gudina et al 2011 | 22 | 100% | High |
| 21 | Tamiru et al 2010 | 18 | 81.8% | High |
| 22 | Gill et al 2008 | 16 | 72.7% | Low |

STROBE: Strengthening the Reporting of Observational Studies in Epidemiology

STROBE Statement—Checklist of items that should be included in reports of ***cross-sectional studies***

|  | Item No | Recommendation |
| --- | --- | --- |
| **Title and abstract** | 1 | (*a*) Indicate the study’s design with a commonly used term in the title or the abstract |
|  |  | (*b*) Provide in the abstract an informative and balanced summary of what was done and what was found |
| Introduction | | |
| Background/rationale | 2 | Explain the scientific background and rationale for the investigation being reported |
| Objectives | 3 | State specific objectives, including any prespecified hypotheses |
| Methods | | |
| Study design | 4 | Present key elements of study design early in the paper |
| Setting | 5 | Describe the setting, locations, and relevant dates, including periods of recruitment, exposure, follow-up, and data collection |
| Participants | 6 | (*a*) Give the eligibility criteria, and the sources and methods of selection of participants |
| Variables | 7 | Clearly define all outcomes, exposures, predictors, potential confounders, and effect modifiers. Give diagnostic criteria, if applicable |
| Data sources/ measurement | 8* | For each variable of interest, give sources of data and details of methods of assessment (measurement). Describe comparability of assessment methods if there is more than one group |
| Bias | 9 | Describe any efforts to address potential sources of bias |
| Study size | 10 | Explain how the study size was arrived at |
| Quantitative variables | 11 | Explain how quantitative variables were handled in the analyses. If applicable, describe which groupings were chosen and why |
| Statistical methods | 12 | (*a*) Describe all statistical methods, including those used to control for confounding |
|  |  | (*b*) Describe any methods used to examine subgroups and interactions |
|  |  | (*c*) Explain how missing data were addressed |
|  |  | (*d*) If applicable, describe analytical methods taking account of sampling strategy |
|  |  | (*e*) Describe any sensitivity analyses |
| Results | | |
| Participants | 13* | (a) Report numbers of individuals at each stage of study—eg numbers potentially eligible, examined for eligibility, confirmed eligible, included in the study, completing follow-up, and analysed |
|  |  | (b) Give reasons for non-participation at each stage |
|  |  | (c) Consider use of a flow diagram |
| Descriptive data | 14* | (a) Give characteristics of study participants (eg demographic, clinical, social) and information on exposures and potential confounders |
|  |  | (b) Indicate number of participants with missing data for each variable of interest |
| Outcome data | 15* | Report numbers of outcome events or summary measures |
| Main results | 16 | (*a*) Give unadjusted estimates and, if applicable, confounder-adjusted estimates and their precision (eg, 95% confidence interval). Make clear which confounders were adjusted for and why they were included |
|  |  | (*b*) Report category boundaries when continuous variables were categorized |
|  |  | (*c*) If relevant, consider translating estimates of relative risk into absolute risk for a meaningful time period |
| Other analyses | 17 | Report other analyses done—eg analyses of subgroups and interactions, and sensitivity analyses |
| Discussion | | |
| Key results | 18 | Summarise key results with reference to study objectives |
| Limitations | 19 | Discuss limitations of the study, taking into account sources of potential bias or imprecision. Discuss both direction and magnitude of any potential bias |
| Interpretation | 20 | Give a cautious overall interpretation of results considering objectives, limitations, multiplicity of analyses, results from similar studies, and other relevant evidence |
| Generalisability | 21 | Discuss the generalisability (external validity) of the study results |
| Other information | | |
| Funding | 22 | Give the source of funding and the role of the funders for the present study and, if applicable, for the original study on which the present article is based |
