# Supplementary figures and images for: Glycemic control among diabetic patients in Ethiopia: A systematic review and meta-analysis
Source: PLoS One. 2019 Aug 27;14(8):e0221790. doi: 10.1371/journal.pone.0221790 (PMC6711596; doi:10.1371/journal.pone.0221790)

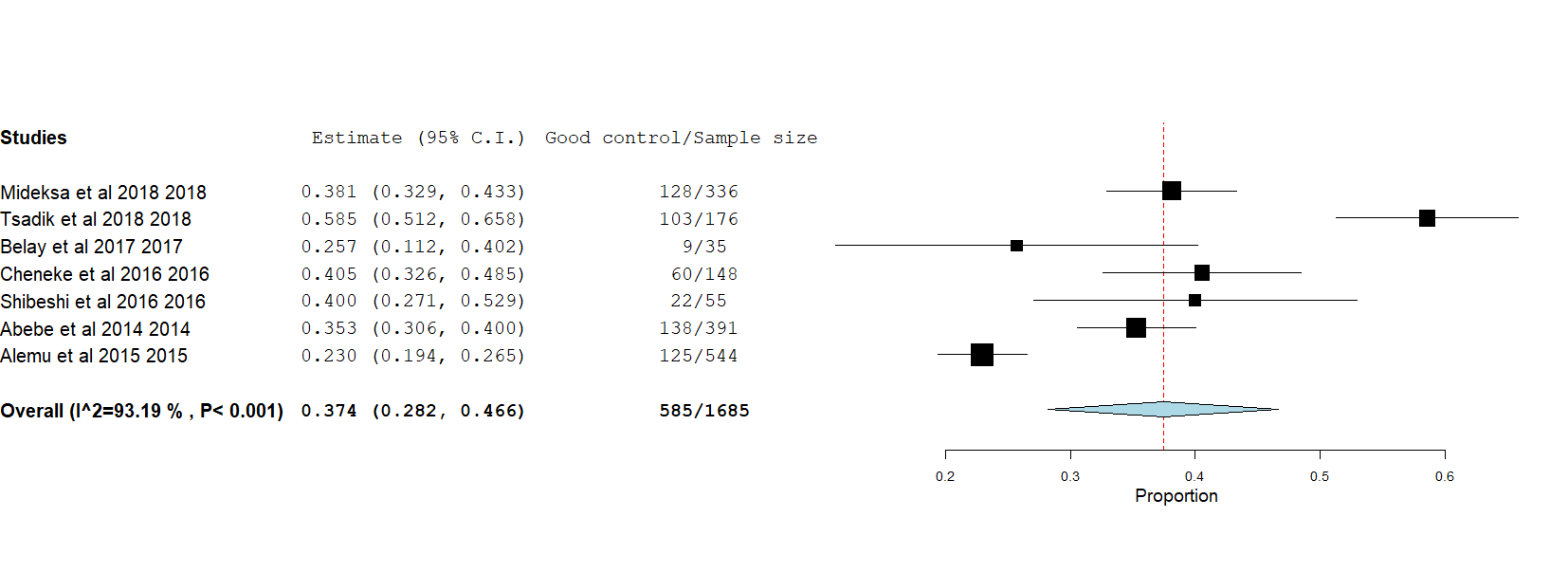

Supplement: S1 Fig — (TIF) [file pone.0221790.s004.tif]

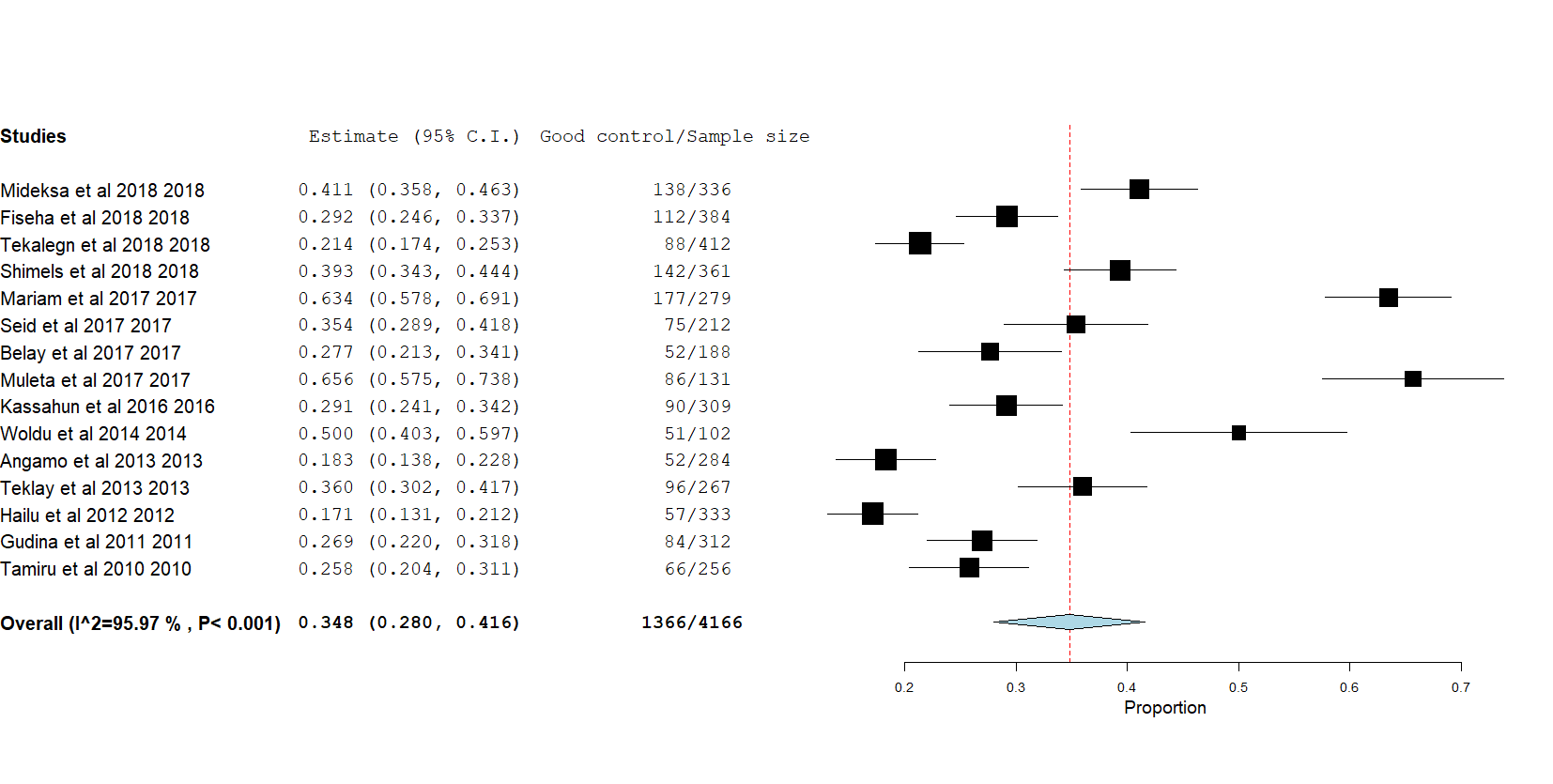

Supplement: S2 Fig — (TIF) [file pone.0221790.s005.tif]
